# A novel holistic metric for sustainability assessment of photovoltaic/battery systems

**DOI:** 10.1038/s41598-025-14809-z

**Published:** 2025-08-16

**Authors:** Rasha Elazab, Mohamed Daowd

**Affiliations:** Faculty of Engineering, Helwan University, Cairo, Egypt

**Keywords:** Photovoltaic, Battery longevity, Energy management systems, HOMER, Sustainability metrics, Load matching, Energy science and technology, Renewable energy, Solar energy, Photovoltaics

## Abstract

The increasing reliance on renewable energy has increased the need for efficient, sustainable energy systems, particularly photovoltaic (PV)-battery systems, which are vital for many applications in diverse climatic regions and directly contribute to SDG 7 (Affordable and Clean Energy). Achieving sustainability in such systems requires evaluating their performance across different locations and climatic conditions, where traditional metrics often fall short in capturing the complexities of energy utilization and battery behavior. This study proposes a novel holistic metric (HM) that incorporates battery performance, energy utilization, and load dynamics, providing a more accurate measure of system performance and supporting SDG 12 (Responsible Consumption and Production). The methodology involves the use of hybrid optimization of multiple energy resources (HOMER) software to simulate PV-battery systems in three locations, namely, Cairo, Berlin, and Riyadh, over a three-year period (2017–2019), with a focus on the battery state of charge (SOC), cycling behavior, and energy efficiency. The results indicate that Riyadh, with its balanced solar conditions, achieved the highest long-term viability (HM ≈ 0.74), followed by Berlin (HM ≈ 0.69) and Cairo (HM ≈ 0.67), with Cairo’s oversized PV system and Berlin’s low solar availability influencing battery performance and system efficiency. This study concludes that tailoring PV-battery system design and energy management strategies to local conditions is crucial for optimizing battery longevity, energy efficiency, and system resilience, addressing SDG 13 (Climate Action). The findings contribute to a more comprehensive approach for evaluating and improving the resilience of PV-battery systems, addressing gaps in conventional sustainability metrics.

## Introduction

Sustainability is a multifaceted concept that addresses the environmental, economic, and social dimensions of energy systems to meet present energy needs while ensuring that future generations can meet those needs^1,2^. As the global transition toward renewable energy has accelerated, photovoltaic (PV)-based systems have gained significant attention because of their ability to provide clean, reliable, and cost-effective energy solutions^3,4^. When coupled with battery energy storage, these systems further enhance energy security and reduce the dependence on fossil fuels. However, ensuring resilience in isolated PV-battery systems requires a holistic evaluation that considers not only energy generation but also battery performance and load dynamics under real-world operational conditions.

A substantial body of research has explored the feasibility and long-term viability of PV-battery systems. Notable studies, such as those by Behar et al.^5^, emphasize the technical and economic advantages of PV integration in industrial and urban contexts.

Similarly, Nassar and Alsadi^6^ investigated the potential of solar energy in challenging environments, with a focus on energy reliability and resource availability. While these studies provide valuable insights, they often focus on isolated aspects of performance, such as energy efficiency or economic feasibility, without fully capturing the complex interactions between PV systems, battery storage, and load demand^7–9^.

Additionally, traditional sustainability metrics, such as loss of load probability and reliability indices, typically overlook the temporal dynamics of load matching, PV energy utilization, and battery performance over extended periods^10,11^.

Furthermore, while metrics such as depth of discharge (DoD), cycle life, and load match index (LMI) are commonly used, they fail to address the operational complexities and variability inherent in renewable energy systems^12^.

The DoD does not account for the long-term degradation caused by fluctuations in the SOC range. Similarly, while the LMI evaluates the alignment of energy supply and demand, it overlooks the critical influence of variable solar irradiance, which directly affects the system’s reliability and operational stability. These shortcomings hinder a more comprehensive understanding of the sustainability of isolated PV systems under real-world operating conditions.

Battery performance is crucial for the overall long-term viability of PV-battery systems, particularly since the state of charge (SOC) significantly impacts battery lifespan. Previous studies^13^ have shown that maintaining the SOC within optimal ranges minimizes capacity fading, thereby extending battery life and improving system profitability. Energy management systems (EMSs), such as HOMERs, aid in optimizing system design and energy dispatch^14,15^. However, the sustainability metrics derived from these tools require refinement to better reflect the dynamic and multifaceted nature of modern energy systems.

Moreover, while environmental indicators such as CO₂ emission reduction^16^ and the renewable energy fraction^17^ offer important perspectives, they are not sufficient on their own to provide a comprehensive assessment of an energy system’s sustainability. Additionally, minimizing operational costs, as discussed in^18,19^, does not necessarily ensure sustainable system behavior, particularly in isolated PV/battery configurations, where the operational costs are minimal and the economic viability largely depends on the longevity and performance of the battery system.

This study proposes a novel holistic metric (HM) for evaluating the performance of isolated PV-battery systems. The HM integrates three critical components: the battery sustainability index (BSI), energy reliability efficiency (ERE), and peak-to-demand matching index (PDM). By using the geometric mean of these components, the metric offers a comprehensive assessment of system resilience. Unlike traditional metrics, HM captures temporal battery performance, energy utilization efficiency, and load-matching dynamics while accounting for the variability of climate conditions and operational complexities over a three-year simulation period via HOMER. This approach provides actionable insights into optimizing system design and enhancing long-term viability.

The proposed holistic metric directly supports key sustainable development goals by promoting improved resource efficiency and enhancing the longevity of battery storage systems. By integrating battery sustainability, energy reliability, and load matching factors, the HM enables more informed design and operation decisions that minimize battery degradation and reduce electronic waste. This alignment advances SDG 7 (Affordable and Clean Energy) by optimizing renewable energy utilization, SDG 12 (Responsible Consumption and Production) by fostering sustainable management of battery resources, and SDG 13 (Climate Action) by facilitating the reduction of greenhouse gas emissions through more efficient and reliable integration of photovoltaic systems. This study presents the following key contributions:

- An innovative metric that combines the BSI, ERE, and PDM to offer a comprehensive evaluation of PV-battery system performance.
- A focus on the temporal dynamics of battery performance, energy utilization, and load matching under varying climate conditions over a three-year simulation period.
- The importance of tailored battery autonomy and system design based on regional solar conditions is emphasized to improve efficiency and longevity.
- Limitations in conventional sustainability metrics can be addressed by integrating factors such as SOC management and load variability.
- This study provides a robust framework for policymakers, system designers, and researchers to optimize PV-battery systems for resilience, adaptability, and long-term viability.

The paper is structured as follows: Sect. 2 details the methodology for developing the holistic metric, including the mathematical formulation of the components. Section 3 presents the cases studied, followed by the results and discussion in Sect. 4 on the implications of the findings. Finally, Sect. 5 concludes the study and outlines directions for future research.

## Holistic sustainability metric

This section introduces a novel sustainability metric developed to evaluate isolated PV systems with energy storage comprehensively. The proposed metric integrates three essential components, battery stability, PV system reliability, and demand‒supply alignment, into a unified index, offering a holistic assessment of system resilience. Each component directly contributes to achieving several United Nations Sustainable Development Goals (SDGs), with interconnected relationships between them.

### Battery term

Battery long-term viability is essential for ensuring efficient, long-term operation in energy storage systems. The SOC stability index (SOCSI) and battery sustainability index (BSI) offer a comprehensive framework to evaluate battery health by considering both operational stability and cycle longevity.

The SOCSI quantifies the proportion of time the battery’s state of charge (SOC) remains within an optimal range, ensuring that the battery operates safely and reduces degradation mechanisms such as lithium plating and electrolyte breakdown. In defining the SOCSI, a threshold range of 20–80% for the SOC was adopted to represent the optimal operating window. This selection is grounded in experimental findings and degradation studies, such as those reported by Qu et al.^20^, which comprehensively analyse lithium-ion battery chemistries under realistic usage. While chemicals such as lithium nickel manganese cobalt oxide (NMC) offer high energy density and are widely used in electric vehicles, they are particularly sensitive to degradation when operating at SOC levels above 80%. Conversely, lithium iron phosphate (LFP) batteries, known for their thermal stability and extended cycle life, exhibit a broader tolerance to high SOC ranges, but even they benefit from avoiding extreme charge states. The 20–80% SOC range provides a conservative, chemistry-agnostic threshold that balances degradation risk, performance, and operational safety, aligning with findings in^20^ that demonstrate faster capacity fades when the SOC extends beyond this range, especially in NMC systems. Thus, the SOCSI definition enhances the relevance of the index across various battery technologies by adopting a scientifically supported and widely practiced SOC window.

The SOCSI is mathematically defined as:

$$\:SOCSI=\frac{Time\:of\:SOC\:in\:Optimal\:Range}{Total\:Studied\:Time}$$

The BSI combines SOCSI with the number of charge cycles (NC), providing a multifaceted sustainability metric:

$$\:BSI={\omega\:}_{1}*SOCSI+{\omega\:}_{2\:}*(1-\frac{{NC}_{used}}{{NC}_{rated\:}})$$

where NC_used_ is the actual number of cycles used over the study period. NC_rated_: Manufacturer-rated cycle life of the battery. $\:{\omega\:}_{1}$and $\:{\omega\:}_{2}$: Weights assigned to SOCSI and NC, respectively ($\:{\omega\:}_{1}$+$\:{\omega\:}_{2}$=1). The term $\:(1-\frac{{NC}_{used}}{{NC}_{rated\:}})$ accounts for the remaining useful life of the battery, emphasizing that fewer cycles relative to the battery’s design capacity indicate better long-term viability.

To assign appropriate importance to each component of the proposed battery sustainability index (BSI), we define the weighting factors $\:{\omega\:}_{1}$ and $\:{\omega\:}_{2}$ on the basis of the relative contributions of the state of charge (SOC) stress and cycle-induced degradation to the overall battery aging process. These degradation contributions are denoted as *D*_*SOC*_ and *D*_*cycle*_, respectively, and the weights are calculated as:

$$\:{\omega\:}_{1}=\frac{{D}_{SOC}}{{D}_{SOC}+{D}_{cycle}}\:,{\omega\:}_{2}=\frac{{D}_{cycle}}{{D}_{SOC}+{D}_{cycle}}$$

A recent large-scale review by Qu et al.^20^ reported that SOC-related factors (e.g., high average SOC and prolonged time near upper limits) account for up to 62–65% of the total capacity degradation in lithium-ion batteries, especially in applications with partial cycling and long idle periods. Furthermore, Collath et al.^12^ showed through aging-aware operational modelling that constraining the SOC operating window led to a 30–40% improvement in battery lifetime, whereas merely minimizing cycling had a comparatively lower effect. On the basis of these findings, we conservatively assign *D*_SOC_=0.6 and *D*_cycle_=0.4, resulting in:

$$\:{\omega\:}_{1}=0.6\:\text{a}\text{n}\text{d}\:{\omega\:}_{2}=0.4$$

These weights reflect the empirically supported dominant role of SOC management over cycling frequency in influencing long-term battery sustainability. Moreover, this approach is flexible and allows easy recalibration for different battery chemistries or usage scenarios on the basis of updated degradation models or field data.

The BSI gives more weight to SOC stability, which is key to preserving battery health over time. A BSI above 0.7 indicates a well-maintained battery with minimal cycling, whereas a value between 0.5 and 0.7 suggests moderate performance, with occasional SOC deviations or higher cycle usage. A BSI below 0.5 typically indicates poor performance due to frequent deep cycling or prolonged operation outside the optimal SOC range.

BSIs offer several advantages over existing sustainability indices by providing a more comprehensive and flexible approach to assessing battery health. Unlike traditional metrics that focus solely on individual aspects of battery performance, such as total cycle count or throughput, BSI integrates both the temporal stability of the SOC and the number of charge cycles. This dual-focus approach allows BSIs to better reflect the operational dynamics that impact the long-term viability of batteries. Additionally, the use of adjustable weights makes BSI highly adaptable, enabling it to prioritize either SOC stability or cycle sustainability depending on the specific needs of the application. This flexibility is a significant improvement over conventional indices, which often fail to capture the nuanced interactions between SOC levels and cycle wear. Furthermore, the BSI provides a clearer understanding of battery performance by combining these factors into a single, easy-to-interpret value, which enhances its practical applicability across diverse energy storage systems.

### PV term

The energy reliability efficiency (ERE) evaluates the reliability of a PV system in delivering energy to meet load demands. It calculates the average fraction of daily PV-generated energy utilized effectively.

$$\:ERE=\frac{1}{n}\sum\:_{i=0}^{n}\frac{Daily\:Energy\:Delivered\:to\:Load}{Daily\:PV\:GeneratedEnergy\:}E$$

where n is the number of studied days.

While common PV metrics assess generation capacity and efficiency, they do not address the alignment between energy generation and load demands. The ERE bridges this gap by considering not only the system’s generation capacity but also its effectiveness in addressing energy needs, providing a more nuanced and practical measure of PV performance.

### Load term

The weighted power demand match (PDM) evaluates how appropriately the system meets demand, prioritizing periods with greater importance, such as peak demand hours.

$$\:PDM=\frac{1}{n}\sum\:_{i=0}^{n}\frac{served\:load\:\:energy\left(t\right)*\omega\:\left(t\right)}{total\:demand\:energy\left(t\right)*\omega\:\left(t\right)}$$

where $\:\omega\:\left(t\right)$ is the weighting factor for each time interval t and where n is the number of days studied.

Traditional metrics such as LMI evaluate demand alignment but fail to prioritize critical time intervals or address temporal mismatches effectively. By incorporating a weighting factor, the PDM focuses on the most critical periods of demand, offering a more realistic evaluation of load matching that reflects real-world energy management challenges.

### Holistic metric HM

The holistic sustainability metric, denoted as HM, integrates three terms, BSI, ERE, and PDM, into a unified sustainability measure:

$$\:HM=\sqrt[3]{BSI*ERE*PDM}$$

**Fig. 1:**
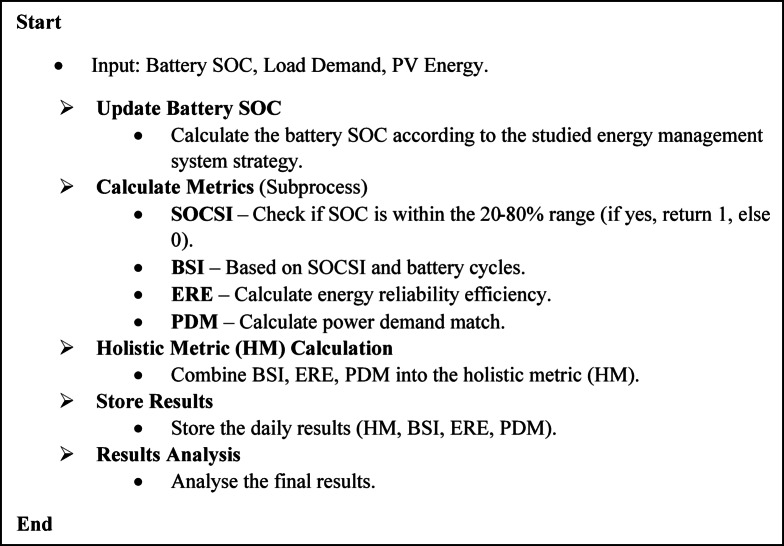
The proposed metric code.

This composite index captures the interdependence of battery stability, PV system reliability, and demand‒supply alignment. By taking the cubic root of the product, the HM ensures balanced contributions from each component, providing a comprehensive evaluation of the system’s long-term viability. This approach not only assesses overall system performance but also reflects the dynamic interactions between these components. The pseudocode of this metric is shown in Fig. 1.

Unlike isolated metrics, the HM offers an integrated evaluation of system performance by addressing critical aspects of operational stability, energy reliability, and demand matching within a single, interpretable index. By incorporating time-based and operational factors, this metric enhances the ability to generate actionable insights for system optimization, especially under variable environmental and demand conditions.

In calculating the proposed HM, the geometric mean is employed to aggregate the three core indices. This method was selected because of its ability to capture the interdependence among normalized, equally weighted dimensions and to penalize imbalance, thereby ensuring that low performance in any one dimension significantly reduces the overall sustainability score. This noncompensatory property is critical in energy systems, where failure in a single subsystem (e.g., battery degradation) can compromise the entire system’s viability. As noted by Singh et al.^21^, the geometric mean is more appropriate than the arithmetic mean or weighted sum in multicriteria evaluations, as it maintains proportional fairness and emphasizes the weakest component, thus offering a more balanced and rigorous assessment of sustainability in complex systems.

The metric’s three-year analysis period offers a robust foundation for assessing the resilience of isolated PV systems. It captures seasonal variations, weather patterns, and climate-related factors, providing a more accurate reflection of system performance over time. This extended timespan enables the identification of long-term trends, variances, and challenges that shorter studies may overlook, ensuring that the metric accounts for fluctuations in solar irradiance, temperature impacts on battery behavior, and changing energy demand.

Additionally, the long-term perspective supports the validation of the EMS, allowing for the assessment of its effectiveness in maintaining system stability and long-term viability. It facilitates the evaluation of degradation rates in key components, such as batteries and PV modules, ensuring that the metric reflects realistic operational conditions. Backed by this comprehensive analysis, the holistic metric strengthens system planning, enhances design, and provides actionable insights for improving the performance of isolated PV systems in diverse and challenging environments.

The proposed holistic sustainability metric, through its components, contributes directly to several SDGs. BSI supports SDG 7 (Affordable and Clean Energy) by ensuring the longevity and efficiency of energy storage systems. The ERE enhances SDG 7 by improving PV system reliability and energy utilization and supports SDG 12 (Responsible Consumption and Production) by optimizing energy use. The PDM promotes SDG 7 by ensuring energy availability during critical demand periods and aligns with SDG 11 (sustainable cities and communities) by enhancing urban energy system resilience.

Together, these components contribute to the HM, which provides a balanced evaluation of battery performance, PV reliability, and demand‒supply alignment. This unified index contributes to SDG 13 (climate action) by fostering energy systems that are resilient to climate impacts. The three-year analysis further supports SDGs 7, 12, and 13 by accounting for long-term climate variability and ensuring the system’s adaptability to changing environmental conditions. Ultimately, the holistic metric offers a robust framework for developing reliable, sustainable, and climate-resilient energy systems.

## Study cases

In this study, the solar irradiance profiles for Cairo, Berlin, and Riyadh were derived for the years 2017–2019 on the basis of standardized datasets provided by the National Renewable Energy Laboratory (NREL)^22^, which identifies them as the most recent continuous period of high-quality, globally consistent irradiance data available across diverse climatic zones. This selection ensures both interregional consistency and temporal reliability, enabling a fair comparison of sustainability performance indicators across the three sites. While a longer temporal span could enhance statistical strength, such an approach would require compromises in data continuity or regional completeness. The chosen period nonetheless captures representative interannual climatic variation, supporting the study’s goals of robust and geographically relevant analysis.

The selection of locations is based on their distinct solar irradiance profiles and climate conditions, as well as their relevance to regional energy strategies. Cairo represents a typical arid region with moderate solar variability and growing solar adoption in Africa and the Middle East. Berlin is a temperate European city with lower irradiance and strong policy-driven renewable integration, whereas Riyadh has a high-irradiance desert climate with extreme operating conditions. These locations were previously employed and validated in a recent peer-reviewed study^23^, which addressed solar uncertainty in microgrid energy management. By spanning different climatic zones, solar potentials, and energy infrastructures, the selected cities allow for a robust comparison of system performance and sustainability metrics across diverse environmental conditions. Future studies may extend the analysis to include coastal or high-altitude regions to further enhance generalizability.

**Fig. 2:**
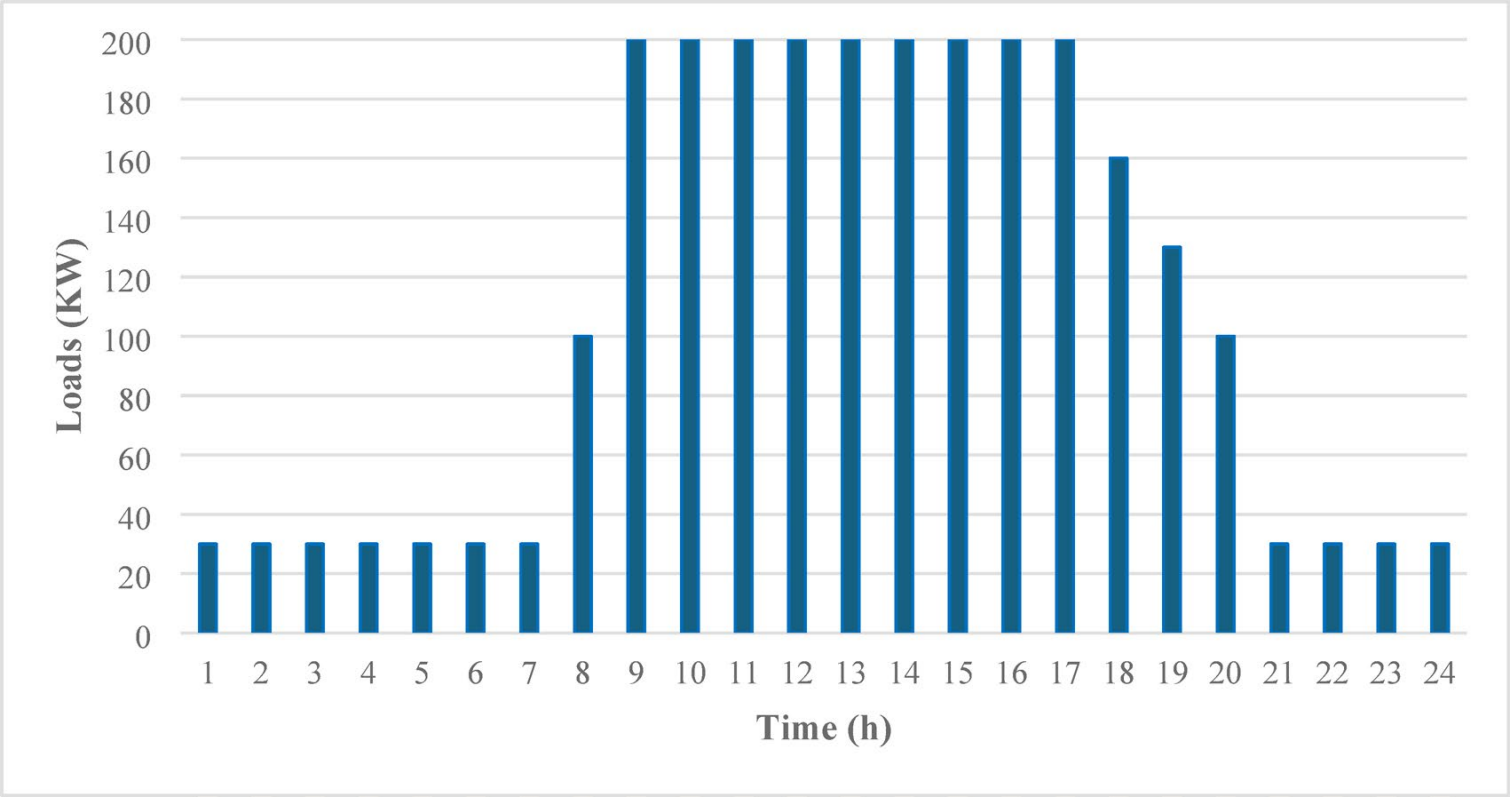
Daily load profile^23^.

The PV system sizes and battery capacities were determined on the basis of HOMER simulations, as discussed in^23^. Figure 2 represents the daily load profile, and the sizes of the PV and batteries are summarized in Table 1.

**Table 1:** PV/Battery system size^23^.

Size	Cairo	Berlin	Riyadh
PV (kWp)	800	1604	769
Generic Li-ion battery (kWh)	2800	3000	2700

The PV size was largest in Berlin at 1604 kWp because of its greater reliance on photovoltaic generation to compensate for lower daily solar irradiance than in Riyadh (769 kWp) and Cairo (800 kWp). The battery capacity, however, remained relatively consistent, with Berlin requiring 3000 kWh and Cairo and Riyadh requiring slightly less, at 2800 kWh and 2700 kWh, respectively. These differences reflect the interplay between local solar resources and load requirements, demonstrating the impact of geographic and climatic factors on system design and performance. HOMER’s load-following strategy is chosen for the EMS to evaluate the interaction behavior between the PV, battery, and load.

The simulation platform used in this study is HOMER Pro, a widely validated and industry-standard tool for technoeconomic modelling of hybrid renewable energy systems. The key system parameters are summarized in Table 2. While HOMER offers strong capabilities for evaluating system sizing, cost analysis, and basic battery cycling behavior, several limitations must be acknowledged that may affect the precision of long-term sustainability assessments.

First, the battery degradation model in HOMER is based solely on the cumulative number of charge/discharge cycles and the associated depth of discharge. It does not account for calendar aging, i.e., the degradation that occurs over time regardless of cycling, which is particularly significant for lithium-ion batteries in low-use or standby scenarios. This omission can lead to optimistic estimates of battery lifespan, especially in systems with extended idle periods or partial cycling patterns. Additionally, HOMER does not model temperature-induced degradation or thermal management effects, further limiting its accuracy in representing battery aging in hot or highly variable climates^23^.

Second, HOMER employs a simplified dispatch model. These dispatch methods assume that generation follows the load as closely as possible without performing dynamic optimization or predictive control. Consequently, the model may fail to capture real-world operational strategies (e.g., advanced battery management systems, predictive scheduling, or demand response) that could enhance system performance or extend battery life.

Despite these limitations, HOMER remains a robust and peer-validated simulation environment. Its use in this study is justified by its wide adoption in similar research contexts and its ability to provide reproducible and comparable baseline results. However, future work could integrate more advanced modelling platforms or cosimulations that capture calendar aging, temperature effects, and dynamic dispatch optimization (e.g., MATLAB/Simulink, Python-based models, or proprietary BMS simulations).

**Table 2:** Key HOMER input parameters and modelling assumptions.

Component	Parameter	Value/assumption	Justification/source
PV	Module efficiency	20%	Typical for commercial monocrystalline panels
	Degradation rate	0.5% per year	Industry average; supported by [21]
	Capital cost	900 $/kW	Based on regional estimates
	Lifetime	25 years	HOMER default; industry average
Battery system	Technology type	Lithium-Ion (assumed)	Widely used in distributed systems
	Round-trip efficiency	85%	Manufacturer specs; consistent with HOMER defaults
	Cycle life	4000 cycles at 80% DoD	Typical for Li-ion cells
	Degradation model	Cycle-based aging only	HOMER limitation; no calendar aging
	Initial SOC	50%	Default assumption
	Min/Max SOC	20%/80%	Based on battery health optimization [22], [23]
Load profile	Load type	Residential	Based on local demand profiles in [21]
	Daily variation	Diurnal (standard HOMER template)	Reflects real-world residential usage
	Seasonal variation	Higher in summer	Climate dependent; aligned with Cairo profile
System dispatch	Strategy	Load Following	HOMER default strategy
	Grid-following behavior	Not applicable (isolated systems)	Assumes off-grid scenario
Inverter	Efficiency	96%	Manufacturer average
Simulation duration	Time span	3 years (2017–2019)	Most recent complete dataset available from NREL [20], [21]

## Results and discussion

In this section, the results of each sustainability metric are individually analysed to examine the dynamics of battery performance, PV power generation, and load behavior. Finally, the holistic metric is evaluated to provide a comprehensive overview of the cases studied and their overall resilience.

### Battery sustainability index (BSI)

In this study, the BSI was designed to provide a computationally tractable representation of battery health by quantifying SOC excursions and cycling frequency via weightings supported by degradation trends in the literature^12,20^. While this formulation captures the operational stress imposed on batteries, it does not include detailed electrochemical models such as Rainflow Counting or calendar aging estimation. For example, in Berlin, the BSI highlighted more frequent low-SOC events—reflecting deeper discharge cycles—which are known to accelerate degradation in both NMC and LFP batteries. These findings are consistent with trends reported in experimental investigations on hybrid energy storage, such as in^24^, where lithium-ion battery degradation was significantly affected by real-world fluctuations in renewable energy output. The incorporation of such empirical modelling approaches and hybrid storage configurations will be a natural extension of this work, particularly to bridge the gap between operational patterns and electrochemical wear.

**Fig. 3:**
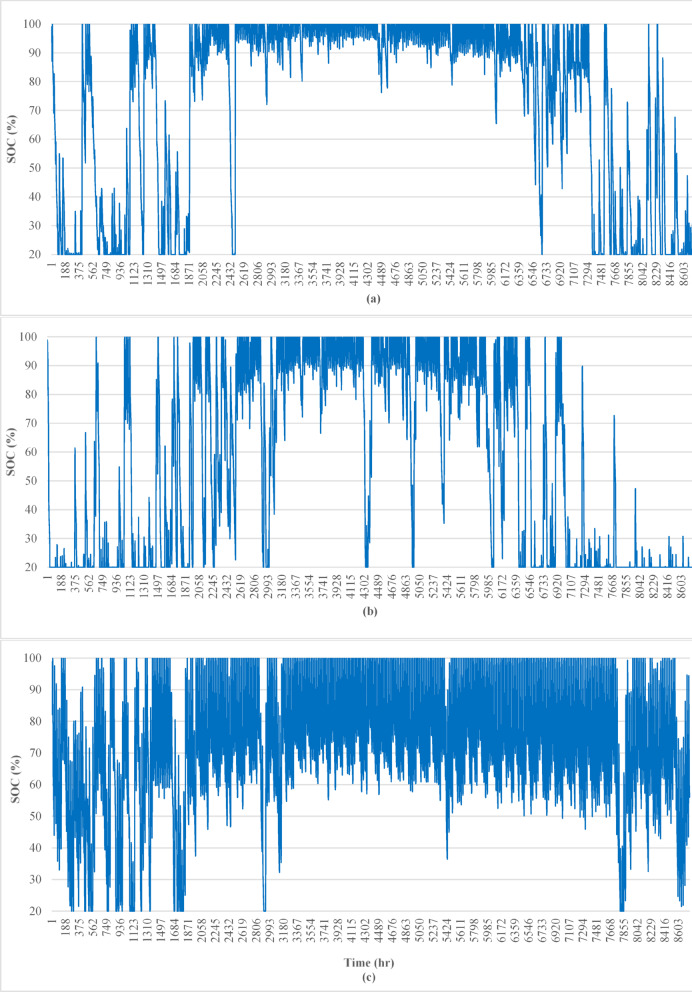
Comparison of state-of-charge (SOC) behavior across Cairo, Berlin, and Riyadh for 2017, illustrating the temporal dynamics and variability influenced by regional solar conditions and battery management.

The SOC is maintained above the minimum threshold of 20% for most of the operational hours, as set by HOMER. However, the frequency and duration of excursions below the safe operating range vary significantly across locations (see Fig. 3). The SOCSI is calculated for all the study cases, as shown in Table 2.

For Cairo (a), the SOC profile indicates that the battery spends most of the year at or near full capacity (100% SOC). This suggests an oversized energy storage system, where PV generation consistently exceeds demand. The frequent operation at full SOC may indicate underutilization of the system’s capacity and potential for optimization to reduce oversizing.

For Berlin (b), the SOC profile shows more variation, with periods of SOC decreasing closer to the 20% threshold. However, the battery generally maintains the SOC within the safe range for a significant portion of the year. The balanced performance reflects a system that is reasonably well sized, although there may still be occasional mismatches between PV generation and demand.

For Riyadh (c), the SOC profile demonstrates the most consistent behavior, with the SOC remaining stable and within the safe range for most of the operational hours. The battery rarely exists at critical levels, which reflects effective PV generation and proper system sizing suited to Riyadh’s high solar irradiance.

The evaluation of the BSI and SOCSI over the three years (2017–2019) reveals significant differences in battery performance due to regional variations in solar energy availability, operational dynamics, and battery sizing, as shown in Table 3.

**Table 3:** Battery sustainability Indices.

SOCSI (fraction)	2017	2018	2019	BSI (fraction)	2017	2018	2019
Cairo	0.383	0.529	0.468	Cairo	0.614	0.702	0.666
Berlin	0.605	0.488	0.559	Berlin	0.755	0.685	0.728
Riyadh	0.711	0.707	0.714	Riyadh	0.807	0.806	0.809

**Table 4:** SOC quantitative indicators.

Location	Cairo			Berlin			Ryadih		
Year	2017	2018	2019	2017	2018	2019	2017	2018	2019
Unsafe time	5408	4125	4658	3461	4487	3862	2535	2562	2509
Mean	72.3	77.8	82.7	56.4	63.4	58.3	70.1	70.2	69.6
Standard deviation	32.1	18.1	11.9	33.8	34.2	34.6	19.6	19.9	20.2

Although Fig. 3 provides insight into the SOC behavior over time, the SOC pattern is highly dynamic and influenced by several interdependent variables, including load demand fluctuations, solar radiation variability, and autonomy configuration. As such, determining the long-term sustainability or degradation risk of a battery on the basis of SOC plots or average values alone may lead to ambiguous conclusions. This complexity highlights the necessity of developing a comprehensive metric that encapsulates not only SOC trends but also their implications for battery longevity and system resilience.

Table 4 presents quantitative indicators of battery SOC behavior across three distinct locations (Cairo, Berlin, and Riyadh) over three years, including the average SOC, standard deviation, and cumulative time the battery operated outside the optimal SOC window. The results reveal significant spatial and temporal variations in battery operating patterns. For example, Cairo presented relatively high average SOC values (72.3–82.7%) with decreasing standard deviations over time (32.1–11.9%), reflecting tighter SOC control but frequent exposure to high SOC levels that risk accelerated degradation. Conversely, Berlin’s SOC remained generally lower (56.4–63.4%), yet with consistently high variability (approximately 34%), indicating less consistent SOC management. Riyadh displayed a stable average SOC (approximately 70%) and moderate standard deviation (≈ 20%), suggesting relatively balanced operation.

The SOCSI results reveal significant differences in battery performance across the studied locations, highlighting the importance of optimizing battery autonomy on the basis of regional solar conditions. In Cairo, an oversized PV system designed to provide one full day of autonomy consistently results in lower SOCSI values. This is because the system generates more power than the load demand does, causing the battery to remain near its maximum SOC for most of the year. While this setup reduces deep discharges, it limits the battery’s cycling within the optimal SOC range, resulting in less dynamic operation and higher degradation risks. Therefore, despite ensuring a reliable energy supply, Cairo’s battery system operates suboptimally in terms of long-term viability, with the BSI reflecting this imbalance between autonomy and SOC management.

However, these conventional statistical measures (mean and standard deviation) fail to capture critical aspects of battery sustainability. For example, both Berlin 2017 and Cairo 2017 show similar standard deviations (33.8% vs. 32.1%), yet the time spent outside the safe SOC range differs drastically (3461 h vs. 5408 h). This illustrates that traditional statistics provide limited insight into the actual stress imposed on a battery. The unsafe time metric highlights these hidden risks, emphasizing the need for a composite index such as the BSI, which integrates both the frequency and severity of SOC deviations alongside cycling behavior. The BSI thus offers a more meaningful and actionable indicator of battery health, supporting informed decisions about system operation and maintenance planning.

In contrast, Berlin experiences higher SOCSI values due to limited solar energy, which requires the battery to operate within a narrow SOC range. The battery spends much of the year at the lower SOC limit of 20%, especially in colder climates. However, the system’s ability to maintain this low SOC range leads to better resilience in terms of cycle life. Berlin’s higher BSI indicates a more favourable balance between cycling frequency and SOC management than does Cairo, where the oversized system limits cycling dynamics.

Riyadh benefits from consistently high solar availability, leading to more balanced and dynamic battery operation. The battery in Riyadh operates within the optimal SOC range more frequently, which promotes both longevity and reduced degradation. Riyadh’s high BSI values underscore its more sustainable battery performance, attributed to its optimal balance between SOC stability and efficient cycling.

The results emphasize the need for location-specific battery operation strategies. In Cairo, the oversized PV system should be optimized to avoid excessive SOC levels, as one day of autonomy is excessive for such sustainable high solar radiation. Conversely, in Berlin and Riyadh, one day of autonomy is more appropriate given the limited solar availability and consistent solar conditions, respectively. These findings highlight that autonomy should be carefully adjusted to local solar power performance, optimizing both battery performance and long-term viability across diverse environmental contexts.

To assess how different degradation mechanisms or battery chemistries influence the BSI, a sensitivity study was conducted where the weights were varied as follows:

- *Scenario A* SOC-dominant scenario (ω₁ = 0.7, ω₂ = 0.3).
- *Scenario B* Base case (ω₁ = 0.6, ω₂ = 0.4).
- *Scenario C* Equal contribution (ω₁ = 0.5, ω₂ = 0.5).
- *Scenario D* Cycle dominant (ω₁ = 0.4, ω₂ = 0.6).

**Table 5:** BSI values under each scenario.

Location	Cairo			Berlin			Riyadh		
Scenario	2017	2018	2019	2017	2018	2019	2017	2018	2019
0.7/0.3	0.556	0.658	0.616	0.717	0.636	0.685	0.783	0.781	0.786
0.6/0.4	0.614	0.702	0.666	0.755	0.685	0.728	0.807	0.805	0.809
0.5/0.5	0.672	0.745	0.715	0.792	0.735	0.77	0.831	0.83	0.833
0.4/0.6	0.73	0.788	0.764	0.83	0.784	0.812	0.855	0.854	0.857

As shown in Table 5, the analysis demonstrates that BSI is responsive to changes in degradation emphasis. For example, in Cairo in 2017, the BSI increased by more than 30% between the SOC-dominant (0.556) and cycle-dominant (0.730) scenarios. This variability highlights the importance of selecting appropriate weights to reflect the actual degradation mechanisms relevant to battery technology and the usage profile.

Lithium-ion chemistries such as NMC, which are widely used in advanced systems, are highly sensitive to SOC-related degradation, justifying higher SOCSI weightings (e.g., ω₁ = 0.6–0.7). In contrast, chemistries such as LFP or future solid-state batteries, which exhibit greater tolerance to high SOC, may benefit from recalibrated weights with greater emphasis on cycling. Similarly, systems with high-frequency shallow cycling may prioritize the cycle term to better capture wear patterns.

This flexibility enhances the applicability of BSI across diverse technologies and operating conditions. The sensitivity results also reinforce the need for careful, data-driven selection of weights rather than arbitrary choices, ensuring that the index meaningfully reflects the sustainability of the battery system.

### PV power behavior

**Fig. 4:**
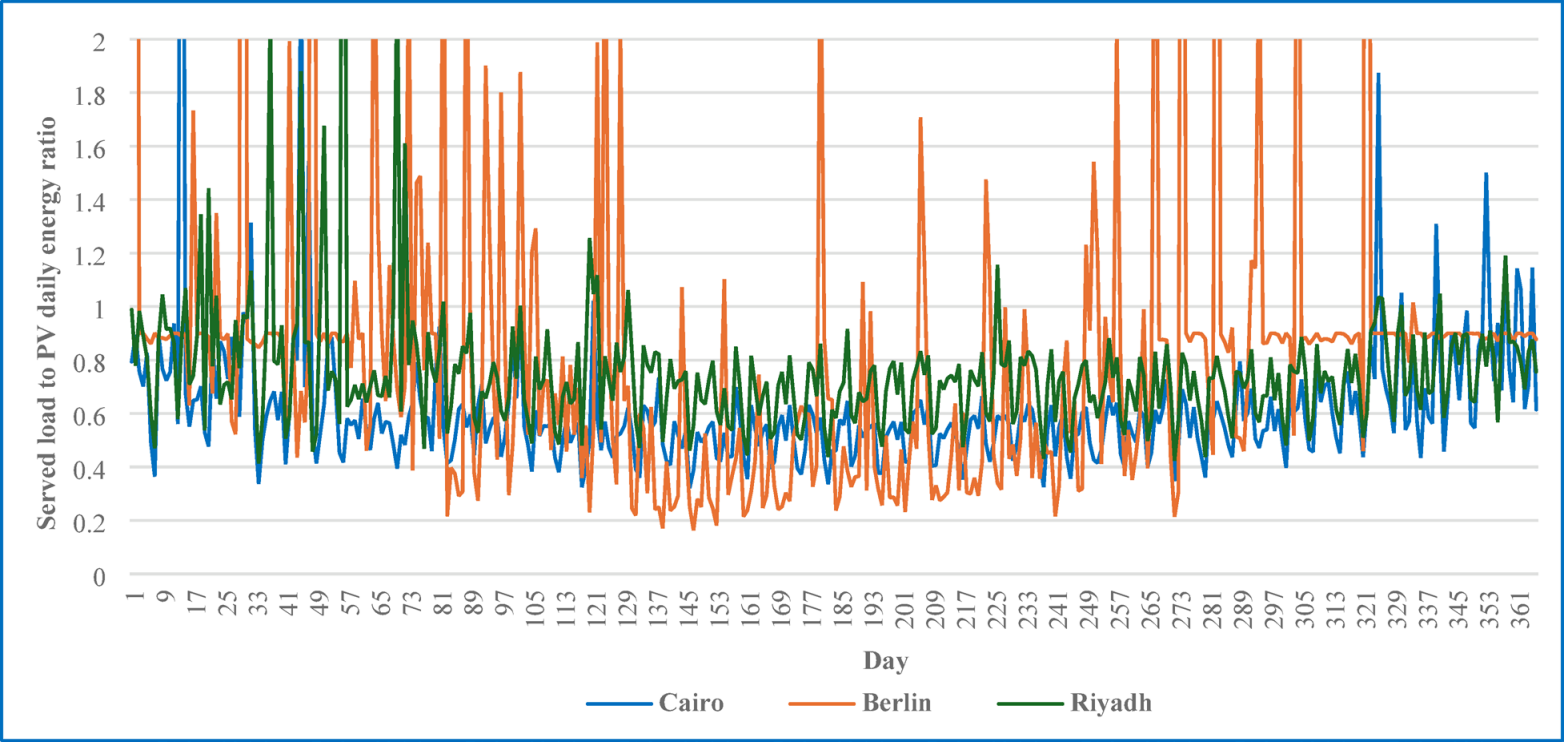
Comparison of the daily ratio of served load to PV energy across Cairo, Berlin, and Riyadh for 2017, showing regional differences in PV system performance and load matching.

Figure 4 shows the daily ratio of served load to PV energy for three cities, i.e., Cairo, Berlin, and Riyadh, over 2017. Cairo consistently has a stable ratio below 1, indicating that PV energy production generally exceeds the load throughout the year. This pattern reflects the effective sizing of the PV system and favourable solar conditions, which minimize seasonal variability and ensure consistent energy availability. Berlin, in contrast, demonstrated significant fluctuations in the ratio, with multiple peaks exceeding 1. These spikes indicate periods where the served load surpasses PV energy production, particularly during winter months with lower solar irradiance. This variability highlights the challenges of relying solely on PV systems in regions with significant seasonal changes in solar availability, necessitating greater reliance on storage. Riyadh displays a pattern similar to that of Cairo, with ratios below 1 for most of the year, although it experiences slightly more variability. Occasional peaks suggest short-term mismatches between PV energy generation and load, but the overall stability reflects Riyadh’s high solar irradiance and well-sized PV system.

Table 6 summarizes the ERE values for the three locations over three years. The ERE evaluates the PV system’s reliability in delivering energy to meet load demands by calculating the average fraction of daily PV-generated energy utilized effectively. Cairo’s ERE values, ranging from 0.55 to 0.63, indicate a consistent and reliable energy supply where PV production typically exceeds load requirements. This reflects a surplus of PV energy, which is effectively utilized to serve the load, minimizing reliance on storage.

Although Berlin has the lowest solar irradiance among the three locations, it presented the highest ERE value in 2017 (0.878). This outcome is attributed not to higher energy availability but to better alignment between the PV system output and the local load profile, resulting in more efficient utilization of the available PV energy. The relatively moderate and consistent load demand in Berlin allows a larger portion of the PV-generated energy to be consumed directly, thereby increasing the ERE despite limited solar resources.

The fluctuation in Berlin’s ERE values across the years, particularly the noticeable drop in 2018 (0.732), can be attributed to seasonal variations in irradiance, which affect the degree of mismatch between generation and load. Unlike Cairo, where PV generation often exceeds load requirements year-round, Berlin’s performance is more sensitive to annual solar variability. These differences highlight the importance of system design and regional climate in determining the effective utilization of PV energy.

Riyadh has an ERE of approximately 0.74, indicating a well-balanced system where PV energy production and load demands align effectively. The consistency of these values highlights Riyadh’s stable solar resources and effective system sizing, which ensures high reliability in energy delivery across all three years. These observations emphasize the importance of tailoring PV systems and energy management strategies to specific regional conditions to optimize reliability and performance.

**Table 6:** Energy reliability efficiency.

Location	2017	2018	2019
Cairo	0.614	0.63	0.551
Berlin	0.878	0.732	0.79
Riyadh	0.756	0.734	0.745

These year-over-year trends underscore the importance of location-specific PV system design and energy management. While Cairo and Riyadh show consistent reliability, Berlin’s variability suggests the need for more robust storage or adaptive load management in regions with less predictable solar patterns.

### Load behavior

The weighting factor was set to 3 during peak demand hours (08:00–16:00), where the load reached 200 kW, and 1 during other hours. This reflects periods of highest grid stress and system vulnerability, ensuring that the PDM metric prioritizes demand-matching performance during operationally critical times.

**Fig. 5:**
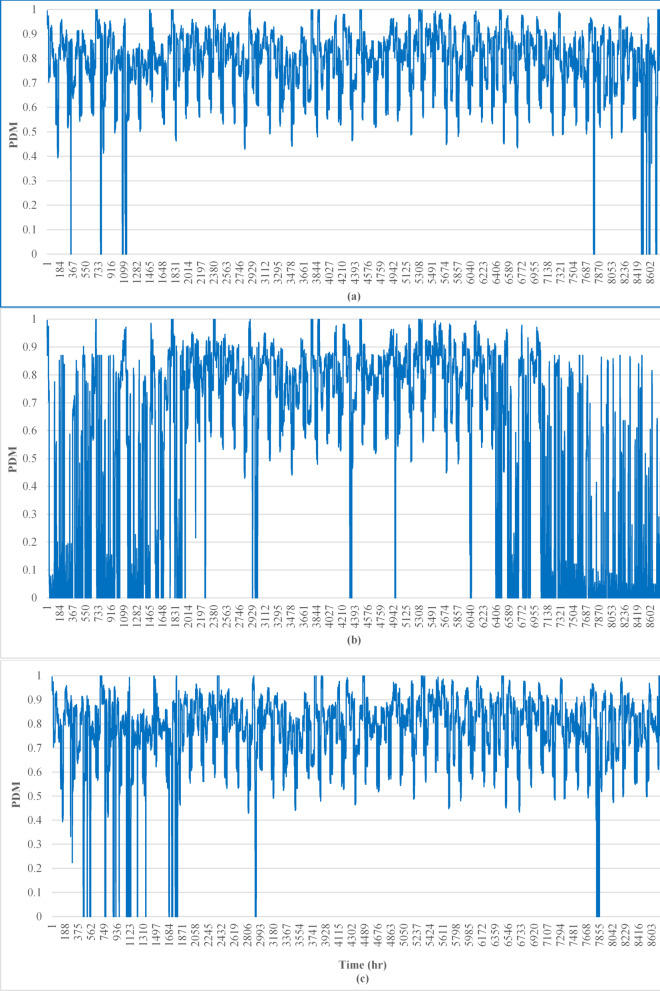
Comparison of the weighted peak demand matching (PDM) metric across Cairo, Berlin, and Riyadh for 2017, reflecting system performance under variable load weighting during peak and off-peak *hours*.

Cairo consistently has high weighted PDM values throughout the study period, with most values remaining above 0.6, as shown in Fig. 5-a. This indicates a well-balanced energy supply and demand, particularly during both peak and off-peak hours. The solar resource availability in Cairo, coupled with an effective EMS, supports stable performance.

Berlin shows a more volatile weighted PDM profile with frequent dips below 0.5 (see Fig. 5-b). This suggests challenges in meeting energy demand, particularly during peak load periods. The lower solar irradiance in Berlin likely impacts the PV performance, and the peak load weight (ω = 3) accentuates this mismatch during high-demand periods.

Riyadh’s weighted PDM values are relatively stable, remaining consistently above 0.5, with fewer extreme dips than those in Berlin. The region benefits from strong solar resources, enabling good performance, as shown in Fig. 5-c. However, occasional drops indicate periods of higher unmet loads, possibly during peak load periods or extreme weather conditions. Table 4 provides further insight into the long-term performance of the metric.

Cairo achieves the highest average PDM across all years. This reflects effective solar PV and EMS practices that ensure a balance between load demand and energy supply. Berlin consistently has the lowest PDM values, highlighting the impact of reduced solar resource availability. Seasonal fluctuations and the higher weight assigned to peak loads exacerbate the challenges in matching energy demand. Riyadh exhibits strong performance, with PDM values close to those of Cairo, as shown in Table 7. Its stable solar irradiance supports effective load balancing, although minor dips indicate areas for improvement in peak load management.

**Table 7:** PDM.

Location	2017	2018	2019
Cairo	0.775	0.774	0.785
Berlin	0.522	0.549	0.519
Riyadh	0.765	0.754	0.757

### Holistic sustainability index

**Fig. 6:**
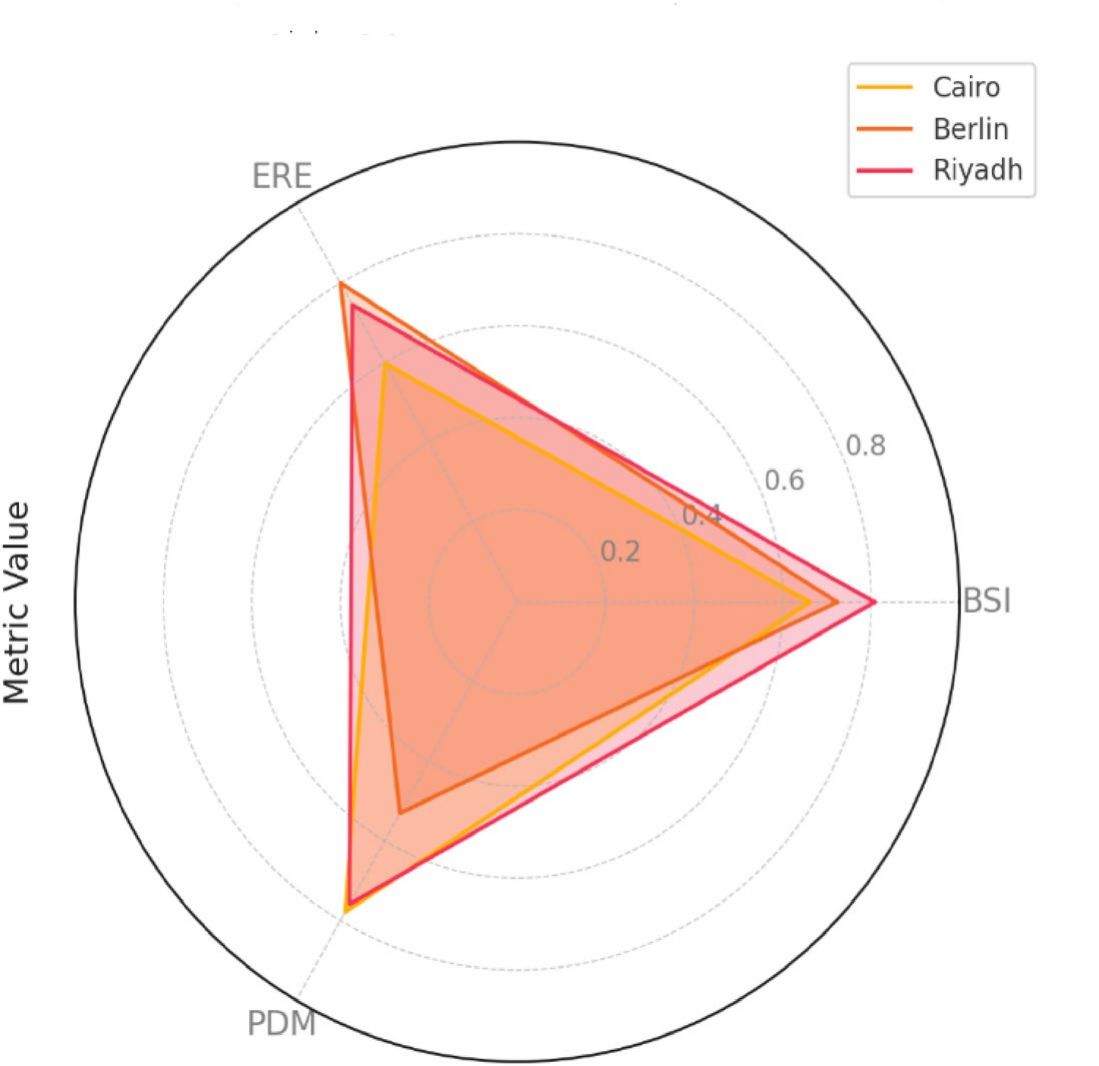
Comparison of BSI, ERE, and PDM metrics for Cairo, Berlin, and Riyadh, illustrating differences in battery sustainability, energy reliability, and demand matching performance across the cities.

The radar chart, shown in Fig. 6, effectively highlights the comparative sustainability performance of Cairo, Berlin, and Riyadh across average values of three key metrics: BSI, ERE, and PDM. Riyadh has the strongest overall sustainability profile, leading to both BSI (0.81) and ERE (0.75), indicating robust battery longevity and efficient energy reliability (see Figure y). Cairo has a relatively high PDM score (0.78), reflecting excellent load matching performance, although its lower BSI (0.66) and ERE (0.60) suggest potential oversizing issues and opportunities for optimizing battery cycling and system efficiency. Berlin, while exhibiting the highest ERE (0.80), has the lowest PDM (0.53), indicating challenges in demand matching, likely due to variable solar availability and seasonal load fluctuations. The intermediate BSI value (0.72) for Berlin indicates moderate battery performance but suggests that system flexibility improvements, such as hybrid storage or renewable diversification, could further enhance sustainability. Overall, the radar chart underscores the distinct trade-offs in system design and operational efficiency among cities, emphasizing the need for tailored strategies to improve sustainability metrics on the basis of regional characteristics.

The final HM values for the three locations, i.e., Cairo, Berlin, and Riyadh, over three years are presented in Table 8.

**Table 8:** Holistic sustainability results.

Location	2017	2018	2019
Cairo	0.664	0.699	0.66
Berlin	0.702	0.65	0.668
Riyadh	0.743	0.7314	0.738

The results of the HM analysis provide insights into the performance of the locations studied over three years. Riyadh achieves the highest HM values across all years, reflecting its optimal balance between battery performance, energy utilization, and load-matching efficiency. Its high solar availability ensures that the battery operates within the optimal SOC range more frequently, promoting longevity while maintaining efficient energy conversion and demand matching.

Berlin has moderate HM values because of its efficient cycling dynamics and relatively strong energy utilization ERE. However, its limited solar availability requires the battery to operate near the lower SOC range for extended periods, increasing stress and degradation risks. While Berlin’s energy management strategy effectively maximizes energy utilization under constrained conditions, improving battery stress management could enhance its overall performance.

Cairo, despite benefiting from an oversized PV system that ensures a reliable energy supply, records comparatively lower HM values. The oversized system results in prolonged periods of high SOC, reducing cycling dynamics and leading to inefficiencies in energy utilization and load matching. Adjusting the system design to better align with local demand patterns and solar availability could significantly improve the performance of Cairo’s HM.

The contrasting system behaviors observed in Cairo and Berlin highlight the need for region-specific optimization strategies. Cairo’s oversized PV system ensures high reliability but leads to suboptimal battery cycling, which lowers the overall HM by underutilizing the battery and potentially reducing its lifespan. Conversely, Berlin’s smaller PV capacity results in greater battery stress due to increased cycling but achieves a higher ERE because of more effective energy utilization. To improve system performance, it is recommended that Cairo’s battery capacity be optimized to increase battery cycling and extend battery longevity. For Berlin, integrating hybrid renewable energy systems, such as combining wind power with PV, could reduce battery stress while maintaining energy reliability. Future work will explore these hybrid configurations to further enhance system flexibility, resilience, and sustainability across diverse climatic regions.

The HM advances beyond traditional reliability-based indices such as the LMI by incorporating a multidimensional assessment of PV-battery system performance. Unlike the LMI, which primarily evaluates how well the load demand is met, the HM integrates the BSI, ERE, and PDM into a unified framework. This integration allows the HM to capture operational inefficiencies and long-term degradation risks that the LMI tends to overlook. A clear example is observed in the Cairo case, where the LMI indicated acceptable reliability performance due to high load coverage, yet the HM revealed a lower score of 0.66, indicating an oversizing issue and excessive battery cycling. This demonstrates HM’s ability to detect subtle but critical design flaws, offering a more insightful evaluation tool for system designers aiming to balance performance, longevity, and reliability.

These findings reinforce the importance of adopting a holistic approach to sustainability assessment, as the interaction between battery performance, energy utilization, and load matching varies significantly across locations. The proposed HM framework effectively captures these interactions, offering actionable insights for optimizing system design and operation to maximize resilience across diverse environmental and operational contexts.

### Economic assessment of PV-battery systems

To enhance the practical relevance of the proposed HM, this section presents a basic economic analysis focused on the levelized cost of energy (LCOE) and battery replacement scheduling. The analysis links sustainability indicators, such as the BSI, to cost performance, highlighting the economic benefits of improved system design (Table 9).

**Table 9:** Economic assumptions and parameters.

Component	Parameter	Value/assumption	Source/justification
PV	Capital cost	900 $/kW	Regional estimate, HOMER default
PV	Lifetime	25 years	Industry average
PV	Degradation rate	0.5%/year	Literature [21]
Battery	Technology	Lithium-ion	Typical for distributed PV systems
Battery	Replacement cost	Same as initial capital	Conservative assumption
Load	Daily load	2620 kWh	Residential profile
Project duration		25 years	Standard for PV systems
Discount rate		6%	Typical for regional studies

#### Levelized cost of energy (LCOE)

The LCOE is calculated as:

$${\text{LCOE }} = {\text{ }}\Sigma {\text{ }}\left( {{\text{C}}_{{\text{t}}} /\left( {{\text{1 }} + {\text{ r}}} \right)^{{\text{t}}} } \right)/\Sigma {\text{ }}\left( {{\text{E}}_{{\text{t}}} /\left( {{\text{1 }} + {\text{ r}}} \right)^{{\text{t}}} } \right)$$

where C_*t*_ is the cost in year t (capital, replacement, O&M), E_t_ is the energy delivered to load in year, r is the discount rate (6%) and T is the project lifetime (25 years).

#### The battery replacement time

The battery replacement time in this study was estimated by linking the BSI results, which reflect the combined effects of SOC stability and cycle usage, to the expected consumption of the battery’s rated cycle life. To link the proposed BSI to battery economic life, we assume a linear relationship between the BSI and the replacement time. This is based on the premise that optimal SOC management and reduced cycling stress, reflected in higher BSI values, enable the battery to approach its maximum potential lifespan. For lithium-ion batteries, a typical maximum calendar life of 15 years is assumed under ideal operating conditions. Thus, the battery replacement time is estimated as:

$$T_{{replace}} = 15 \times BSI$$

When this method is applied, the estimated battery replacement times for Cairo, Berlin, and Riyadh are approximately 9, 13, and 14 years, respectively. These estimates align with practical expectations for lithium-ion battery deployments in well-managed off-grid systems. This approach integrates the technical sustainability analysis with economic planning by translating the BSI into an expected replacement timeline. The economic analysis conservatively assumes a minimum of one replacement during a standard 25-year system lifetime to reflect calendar aging and real-world degradation mechanisms. This integration of sustainability results with replacement scheduling ensures that the proposed metric not only assesses technical health but also informs practical cost planning.

**Table 10:** Economic analysis results.

Location	LCOE ($/kWh)	Estimated Replacement Time
Cairo	0.23	9
Berlin	0.2	13
Riyadh	0.19	14

As shown in Table 10, this basic cost analysis reveals that systems with better battery sustainability (high BSI) not only perform better technically but also reduce long-term costs. In particular,

- Cairo’s frequent cycling (lower BSI) leads to earlier replacements and higher LCOE.
- Riyadh demonstrated that effective SOC management can extend battery life and reduce costs.

By linking HM to LCOE and the replacement frequency, this study provides a comprehensive view of both the technical and economic performance.

## Conclusion

In this study, a novel holistic metric (HM) is proposed to comprehensively evaluate the performance of PV-battery systems by integrating three critical dimensions: the battery sustainability index (BSI), energy reliability efficiency (ERE), and peak-to-demand matching index (PDM). These metrics collectively address battery performance, energy utilization efficiency, and load-matching capability, providing a robust framework for assessing system resilience in diverse environmental contexts. The analysis, which was conducted for Cairo, Berlin, and Riyadh over three years, highlights the interplay of solar conditions, system design, and battery autonomy in shaping performance outcomes.

The results indicate that Riyadh consistently achieves the highest HM values because of its well-balanced performance across all dimensions. High solar availability ensures dynamic battery cycling, optimal SOC operation, and strong load-matching capability, leading to superior resilience. Berlin demonstrates moderate HM values, benefiting from efficient energy utilization and cycling dynamics under limited solar resources. However, prolonged operation at low SOC levels is driven by solar scarcity. Cairo, despite its reliable energy supply supported by an oversized PV system, has comparatively lower HM values because of suboptimal battery cycling and a mismatch between energy generation and load demand, resulting in prolonged high SOC levels and less efficient load matching.

The integration of the BSI, ERE, and PDM in the HM framework underscores the importance of evaluating PV-battery systems from multiple perspectives. While the BSI captures the battery’s operational sustainability, the ERE reflects the system’s ability to efficiently convert available solar energy into usable output, and the PDM assesses the alignment of energy generation with demand patterns. Together, these metrics provide a comprehensive lens for understanding and improving system performance.

Tailoring system design for regional solar conditions has emerged as a critical recommendation. In Cairo, reducing the autonomy of the oversized PV system can address issues related to prolonged high SOC levels and improve energy utilization efficiency. Berlin’s performance could benefit from measures to alleviate battery stress caused by extended low SOC operation, whereas Riyadh’s configuration serves as a benchmark for achieving optimal performance by aligning system parameters with consistent solar conditions.

This research contributes to the field by introducing a scalable and adaptable sustainability assessment framework. Policymakers, system designers, and stakeholders can use this framework to optimize the design and operation of PV-battery systems, balancing operational reliability with long-term viability.

As a direction for future research, the proposed holistic metric (HM) framework can be extended to assess hybrid storage configurations that integrate batteries with complementary technologies such as supercapacitors or hydrogen storage. These hybrid systems offer improved flexibility, power handling, and energy capacity, especially under high-variability or fast-response scenarios. The incorporation of such technologies into the HM framework would enable a more comprehensive evaluation of system resilience and sustainability under diverse operational conditions.

## Data Availability

The datasets used and/or analysed during the current study available from the corresponding author on reasonable request.
